# Fluidized Bed Membrane Reactors for Ultra Pure H_2_ Production—A Step forward towards Commercialization

**DOI:** 10.3390/molecules21030376

**Published:** 2016-03-19

**Authors:** Arash Helmi, Ekain Fernandez, Jon Melendez, David Alfredo Pacheco Tanaka, Fausto Gallucci, Martin van Sint Annaland

**Affiliations:** 1 Chemical Process Intensification, Department of Chemical Engineering and Chemistry, Eindhoven University of Technology, P. O. Box 513, 5612 AZ Eindhoven, The Netherlands; A.Helmi@tue.nl (A.H.); ekain.fernandez@tecnalia.com (E.F.); M.v.SintAnnaland@tue.nl (M.v.S.A.); 2 Energy and Environment Division, TECNALIA, Mikeletegi Pasealekua 2, 20009 San Sebastián-Donostia, Spain; jon.melendez@tecnalia.com (J.M.); alfredo.pacheco@tecnalia.com (D.A.P.T.); 3 Chemical Engineering and Environmental Department, University of the Basque Country UPV/EHU, C/Alameda Urquijo s/n, 48013 Bilbao, Spain

**Keywords:** fluidized bed membrane reactor, water gas shift, ultra-pure H_2_, PEM fuel cell

## Abstract

In this research the performance of a fluidized bed membrane reactor for high temperature water gas shift and its long term stability was investigated to provide a proof-of-concept of the new system at lab scale. A demonstration unit with a capacity of 1 Nm^3^/h of ultra-pure H_2_ was designed, built and operated over 900 h of continuous work. Firstly, the performance of the membranes were investigated at different inlet gas compositions and at different temperatures and H_2_ partial pressure differences. The membranes showed very high H_2_ fluxes (3.89 × 10^−6^ mol·m^−2^·Pa^−1^·s^−1^ at 400 °C and 1 atm pressure difference) with a H_2_/N_2_ ideal perm-selectivity (up to 21,000 when integrating five membranes in the module) beyond the DOE 2015 targets. Monitoring the performance of the membranes and the reactor confirmed a very stable performance of the unit for continuous high temperature water gas shift under bubbling fluidization conditions. Several experiments were carried out at different temperatures, pressures and various inlet compositions to determine the optimum operating window for the reactor. The obtained results showed high hydrogen recovery factors, and very low CO concentrations at the permeate side (in average <10 ppm), so that the produced hydrogen can be directly fed to a low temperature PEM fuel cell.

## 1. Introduction

With the still increasing consumption of fossil fuels and the associated rising energy costs, renewable energy sources are becoming more and more important. Different renewable energy sources are already in use like solar, wind, hydro, *etc.*, but this is not enough to completely replace fossil fuels in the short term. Hydrogen as an energy carrier is widely considered to play an important role in the near future. High purity hydrogen can be used in fuel cells as a substitute for conventional internal combustion engines and gas turbines because of, for example, higher power density and cleaner exhausts [1].

Hydrogen is nowadays mostly used in oil refineries or to produce methanol and ammonia, while the demand for hydrogen is rising. Hydrogen can be produced from different feedstocks, fossil based such as natural gas or coal and non-fossil based such as biomass [2]. The conventional large scale hydrogen production process consists of mainly two steps, the processing of the feedstock (for example reforming or gasification) and the water gas shift (WGS) reaction to upgrade CO to H_2_. The basic reactions for hydrogen production from natural gas (primarily methane) are as follows:

Endothermic reforming of methane

(1) $$CH_{4}+H_{2}O\;↔\;3H_{2}+CO\;\Delta H_{298}^{{}^{\circ}}=+205.9\text{ kJ}/\text{mol}$$

Exothermic water gas shift reaction

(2) $$CO+H_{2}O\;↔\;H_{2}+CO_{2}\;\Delta H_{298}^{{}^{\circ}}=-41\text{ kJ}/\text{mol}$$

The WGS reaction is a very important step in this conversion as it converts carbon monoxide with steam to hydrogen and carbon dioxide. By using a two-stage WGS system with two different catalysts, CO can be almost fully converted reducing its content to values as low as 0.5%.

Indeed, the WGS reaction is an exothermic equilibrium-limited reaction, where the CO conversion and H_2_ production are favored at lower temperatures as can be deduced from the temperature dependency of the equilibrium constant ($K_{p}$) [3]:

(3) $$K_{p}=exp\left(\frac{4577.8}{T}-4.33\right)$$

The WGS reaction is however controlled by kinetics at low temperatures, which implies that a highly active and stable catalyst is required. The WGS reaction is traditionally carried out in a two-stage reactor. This allows a smaller adiabatic temperature rise and a better steam management making the process more economical. The first stage is a high temperature (300–450 °C) converter for fast CO conversion while minimizing the catalyst bed volume. The next stages are at lower temperatures (190–250 °C) to achieve a higher conversion, which is limited by the reaction equilibrium, *i.e.*, temperature and feed composition. Afterward, the produced hydrogen needs to be purified (Pressure swing adsorption (PSA) or Preferential Oxidation (PROX) are the most often used).

The WGS system could be improved by integrating the reaction and separation of hydrogen steps in a single stage [4]. The combination of membrane separation and WGS in membrane reactors have become very interesting, since the membrane separation makes it possible to continuously remove hydrogen which allows conversions beyond the equilibrium conversion of conventional systems thus achieving complete CO conversion in a single high-temperature unit [2,5].

Different types of membrane reactors have been proposed in the literature such as packed bed membrane reactors and fluidized bed membrane reactors. The simplest membrane reactor configuration for WGS is a packed bed membrane reactor where one or more membranes are accommodated in a packed catalytic bed. While this configuration has been proven at lab scale by different authors, still some limitations hamper the application of such configuration when applying highly permeable membranes [4]. In particular, for exothermic reactions, hot spot formation inside the bed (due to the relatively poor heat dispersion in packed bed reactors) can cause damage to the membranes resulting in a decrease in the perm-selectivity and catalyst performance. Additionally, mass transfer limitations from the catalyst bed to the surface of the membrane may prevail when using highly permeable membranes, with the consequent increase in the required membrane area for a given hydrogen recovery [5,6,7].

**Figure 1:**
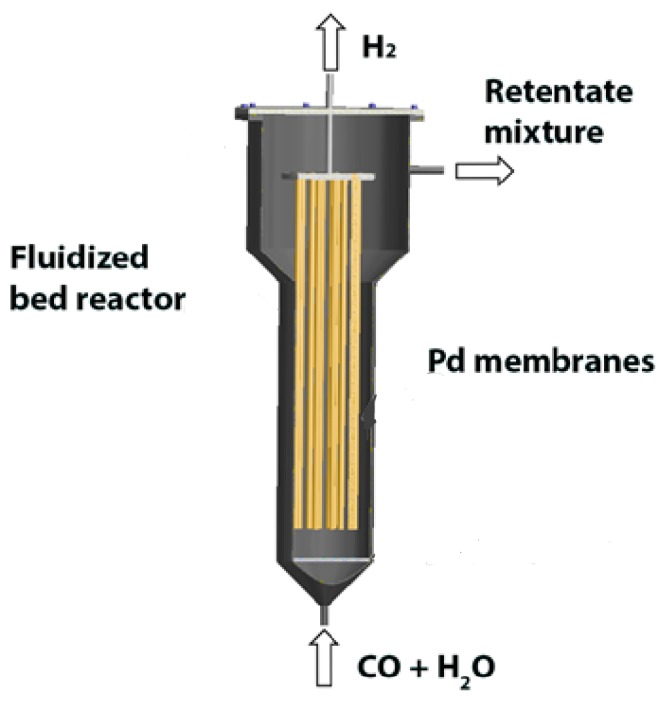
A schematic representation of a fluidized bed membrane reactor for water gas shift reaction.

To overcome the limitations of packed bed membrane reactors, membrane assisted fluidized beds have been proposed in the literature and are being used mostly for methane reforming reactions. In Figure 1, a schematic overview of a fluidized bed membrane reactor for WGS is shown. At the bottom, the reactants are fed to the reactor where the reaction takes place. Palladium (Pd) based membrane tubes are immersed inside the catalyst bed to extract the hydrogen from the reaction zone. To have a successful industrial implementation of such a unit, it is crucial to minimize the required membrane area and the Pd membrane layer thickness for a specific H_2_ production capacity, related to the very high cost of Pd and its scarcity in nature [8].

Negligible pressure drop, reduced intra particle mass and heat transfer resistances, nearly isothermal operating conditions and flexibility in membrane module arrangement are the main advantages of the fluidized bed configuration. In addition, the membrane module can improve the bubble to emulsion mass transfer rate due to increased bubble breakage at the membrane module [9].

Difficulties in reactor construction, membranes sealing and long term stability under fluidizing conditions and erosion of reactor internals and catalyst attrition are the main disadvantages reported for fluidized bed membrane reactor applications, which has thus far hampered the commercialization of these units.

Although membrane assisted fluidized bed reactors have been demonstrated for methane reforming and autothermal reforming reactions with metallic supported Pd-based membranes [10,11], the long term performance of these promising reactors have not yet been demonstrated for WGS using highly permeable ceramic supported Pd-based membranes with high hydrogen recovery factors and selectivities. Therefore, the main objective of this research is to demonstrate a membrane assisted fluidized bed reactor with the capacity of 1 Nm^3^/h of ultrapure H_2_ production with high-temperature WGS to provide a proof-of-principle at lab scale. The long-term performance of the membranes (permeance and selectivity) and the catalyst is monitored in a lab scale test rig. Subsequently, the performance of the membrane reactor is assessed for different inlet compositions and operating conditions to determine the optimum operating window for the process. In the following sections, first results will be presented and discussed, followed by a summary of the main conclusions. Then the experimental setup and procedure is described in detail, discussing practical challenges and possible solutions.

## 2. Results and Discussion 

### 2.1. Conventional Fluidized bed Reactor

**Figure 2:**
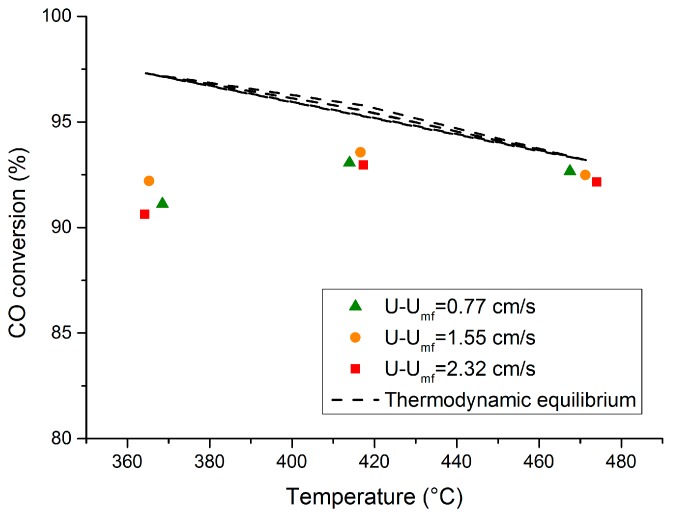
Performance of the conventional fluidized bed reactor a function of the operating temperature at different excess velocities (U-U_mf_) for WGS. Feed: CO (10%), H_2_O (30%), N_2_ (balance), reactor at 1 bar.

Figure 2 illustrates the performance of the catalytic fluidized bed reactor (without membranes) at different temperatures and at different excess velocities (*i.e.*, different U-U_mf_ values, where U is the fluidization velocity and U_mf_ the minimum fluidization velocity) to have a similar initial equivalent average bubble size at different temperatures. According to the obtained results, the performance of the reactor was mainly limited by thermodynamics, where a CO conversion close to the equilibrium value was achieved, especially at higher temperatures. In addition, at constant temperature, the performance of the reactor was similar for different excess velocities, since the CO conversion is not limited by the mass exchange rate between the emulsion and the bubble phases. Therefore, increasing the inlet flow rate will not result in higher CO to H_2_ conversion rates.

Later the performance of the conventional fluidized bed reactor will be compared at similar conditions with the performance of a fluidized bed reactor in the presence of internals and in the last step in the presence of membranes with extraction. After the conventional fluidized bed reactor test, the reactor was cooled down and the catalyst batch was removed from the reactor. Therefore, the membrane module could be integrated for activation protocol under pure H_2_ flow.

### 2.2. Membrane Permeation Properties

In order to characterize the permeation properties of the membranes, initially the membranes have to be activated. The activation protocol includes the reduction under pure H_2_ while imposing a pressure difference between the retentate and permeate sides of the membranes (in this case at 400 °C and 1 bar pressure difference) followed by air treatment of the surface of the membranes (at the same condition). After reaching to a stable performance of the membranes the permeation properties of the membranes were characterized at different temperatures and pressure differences. Table 1 compares the permeation properties of some of the best reported membranes in the open literature with the performance of the used membrane module. Comparing with the reported values, the used membrane module shows a very high H_2_ permeance that can ensure very high H_2_ recovery values. In addition, due to the excellent H_2_/N_2_ ideal selectivity obtained a very high H_2_ purity suitable for low temperature fuel cell applications can be achieved in the permeate side.

To have a fair prediction of the membranes performance at WGS conditions, it is essential to investigate the behavior of the membranes in the presence of WGS related gases. More specifically, the poisoning effect of CO and the inhibitory effect of H_2_O should be quantified. In addition, the external mass transfer limitations for H_2_ molecules to reach the membrane surface have to be taken into account. Therefore, a series of experiments were designed to independently investigate the effects of external mass transfer limitations, CO poisoning and H_2_O inhibition on the permeation properties of the membrane module for different gas compositions at different temperatures.

**Figure 3:**
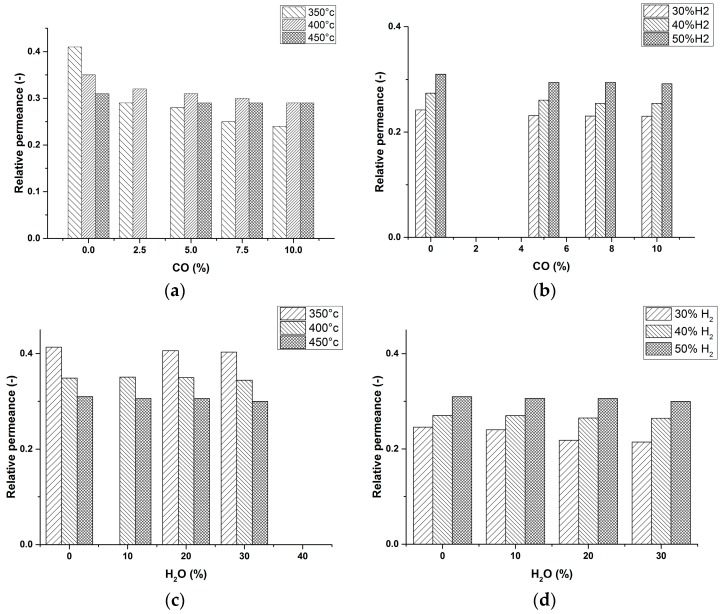
CO poisoning and H_2_O inhibitory effect on the performance of the membranes at different temperatures with H_2_/CO/N_2_ and H_2_/H_2_O/N_2_ feed gas compositions. (**a**) CO poisoning effect at different temperatures and constant H_2_ concentration in feed (50%), N_2_ (balance); (**b**) CO poisoning at different H_2_ concentration in the feed at 450 °C; (**c**) H_2_O inhibitory effect at different temperatures and constant H_2_ in the feed (50%), N_2_ (balance); (**d**) H_2_O inhibitory effect at different H_2_ concentration in the feed at 450 °C. Relative permeance is the performance of the membrane module at the specified condition normalized with the case when only pure H_2_ was used with exactly the same partial pressure difference.

The poisoning effect of CO was quantified by adding different percentages of CO (2.5%–10%) to a mixture of H_2_ and N_2_ with different compositions and at different temperatures (N_2_ was used as the balance). Inspecting the obtained results shown in Figure 3a,b, it can be inferred that, for all cases, CO had a poisoning effect on the performance of the membranes. It can be seen that the poisoning effect of CO is more pronounced at lower CO concentrations. This is in line with the current knowledge on CO interaction with the membrane surface. In fact, CO can easily jump between the hollow and the bridge positions of the Pd cluster, and this permits a small amount of CO to inhibit the interaction of H_2_ with a large part of the Pd surface [26]. At higher temperatures, a less pronounced CO poisoning effect was also observed, as has already been reported by many authors [27]. The possible inhibitory effect of H_2_O on the performance of the membrane was studied as well by addition of certain percentages of H_2_O (30%–50%) to a mixture of H_2_ and N_2_ with different compositions and at a temperature range of 350–450 °C (Figure 3c,d). The addition of H_2_O had a negligible effect on the hydrogen permeance of the membranes.

According to the obtained results (Figure 3a–d), the external mass transfer for H_2_ molecules to reach the membranes surface is affecting the H_2_ permeation flux and this effect is more pronounced for higher temperatures and lower H_2_ concentrations inside the reactor. This is due to the fact that at higher temperatures the membrane has a higher permeance increasing the concentration gradient from the gas bulk to the surface of the membranes (larger extent of concentration polarization). This can be improved by employment of fluidizing particles inside the reactor. Due to a much better mixing of the gas mixture due to solids circulation patterns, the external mass transfer resistance will be largely decreased [19]. Although when immersing very high flux membranes, a dynamic zone with a locally higher solids holdup could be formed (densified zones) in the vicinity of the membranes, which may impose a mass transfer resistance for H_2_ to reach the membrane surface. To mitigate this effect, use of larger particles or operation in the turbulent fluidization regime could to be considered [28].

### 2.3. Long Term Membrane Performance

**Figure 4:**
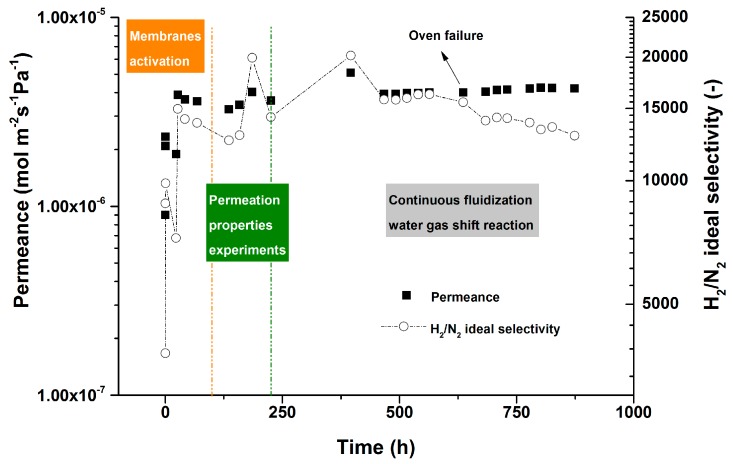
Long-term performance of the membrane module during 900 h of continuous operation in the bubbling fluidization regime at high-temperature WGS conditions. Temperature: 400 °C, P_permeate_: 1 bar, P_retentate_: 2 bar; feed: 10 NL/min of pure H_2_.

After characterization of the membranes permeation properties, the module was cooled down to room temperature and the catalyst batch was integrated inside the reactor. The performance of the membrane module was investigated in the presence of the catalyst in the continuous bubbling fluidization regime at high temperature WGS conditions. Figure 4 summarizes the long-term performance of the membrane module under the specified conditions.

The membrane module showed a very stable performance during nearly 900 h of continuous operation in the bubbling fluidization regime at high temperature WGS conditions (including the initial time used for membrane activation). For such thin and high flux membranes, this is one the longest ever reported stability tests in the literature with an outstanding H_2_/N_2_ selectivity throughout the testing period. Although after 550 h work due to a failure in one of the ovens around the reactor, the membrane module experienced a high thermal shock over night increasing the N_2_ leakage somewhat. This confirms the importance of nearly isothermal conditions to achieve stable performance of the membranes, since hot spot formation by the membranes (which is prone to occur in packed bed membrane reactor modules) could be detrimental for the lifetime and perm-selectivity of the membranes.

### 2.4. Fluidized Bed Membrane Reactor Performance

For all sets of experiments a standard operating procedure was followed to maintain consistency and comparability between the results. First of all, the reactor was set at a desired temperature to be stabilized. Three temperature sensors at the top, middle and bottom of the catalytic bed along the membrane module were placed to continuously monitor the ovens and reactor temperatures to be at the specified set points. Before starting with each experiment, the inlet gas mixture was bypassed to the analyzer to measure the inlet dry gas composition. After inlet gas stabilization in the bypass mode, the feed gas was redirected to the reactor while the membranes were blocked in the permeate side to avoid H_2_ permeation through the membranes as is the case in the conventional fluidized bed reactor (in this case with internals). In the next step, the membranes were opened from the top part and a vacuum pump was used to generate the trans-membrane pressure difference to investigate the effect of H_2_ permeation on the performance of the reactor. Table 2 illustrates the operating window in which the experiments were performed. In the following section, results from the experiments are plotted and discussed in terms of the main reactor performance characteristics, viz. CO conversion and H_2_ recovery factor, defined in Table 3. In addition, for all sets of the experiments the carbon balance was checked to ensure that carbon deposition was always below 2%.

### 2.5. Long Term Performance of the Membrane Reactor

**Figure 5:**
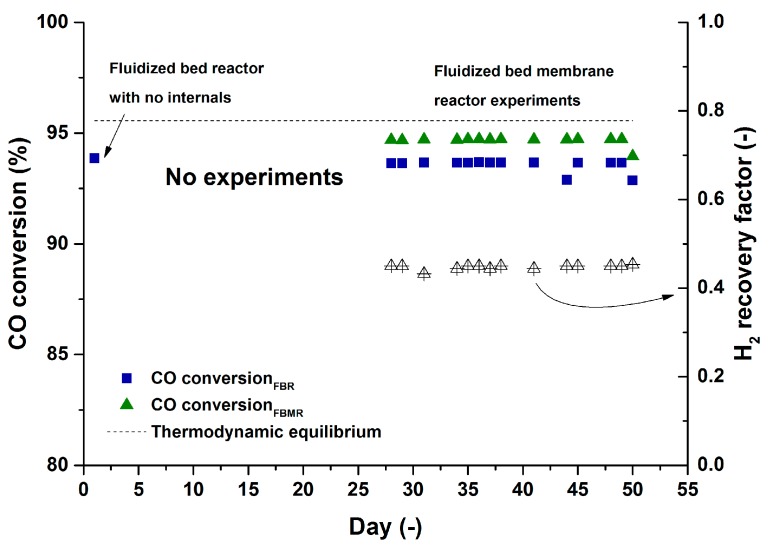
The long term performance of the a fluidized bed membrane reactor (FBMR) module in comparison with a fluidized bed reactor (FBR) module over 900 h of continues work. Temperature: 400 °C, CO (10%), H_2_O (30%), N_2_ balance, U/U_mf_: 2.1, P_perm_: 30 mbar, P:1 bar.

The long term performance of the membrane reactor module was monitored for a base case during the 900 h of continuous operation. The reactor performance was monitored for the base case initially with the membranes closed (a fluidized bed the with membrane module only as internals) and later the membranes were opened in the permeate side to have permeation through them. Figure 5 depicts the performance of the catalyst and membrane reactor over the specified time and for both the fluidized bed reactor (FBR) and fluidized bed membrane reactor (FBMR) cases.

Over roughly 900 hours of continuous operation in the bubbling fluidization regime and WGS operating conditions, the catalyst and the membrane module have shown a very stable performance without any decrease in the performance of the catalyst and permeation properties of the membranes. The CO impurity of the permeate stream was 15 ppm in average during the whole time span (min: 10, max: 28). It should be noted that the CO impurity depends on the CO conversion at the retentate side, so that much lower impurities can be obtained at higher CO conversions at the retentate side (see next section).

A H_2_ recovery factor of 45% in average was measured for the reference case during the experimental work over 900 h. The recovery factor can be either enhanced with increasing the partial pressure of H_2_ at the retentate side (working at elevated pressures), installing more membrane area or increasing the operating temperature of the module which will be explained in the next part of result and discussion.

### 2.6. Fluidized Bed Reactor vs. Fluidized Bed Membrane Reactor

**Figure 6:**
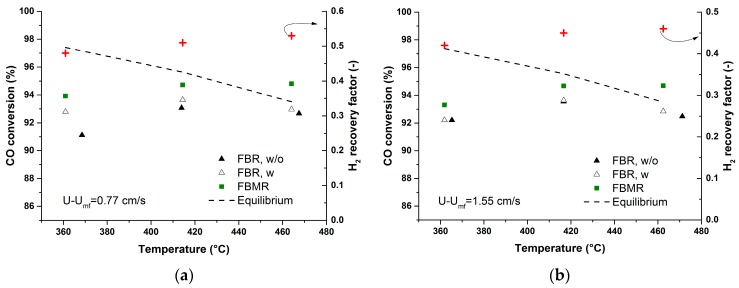

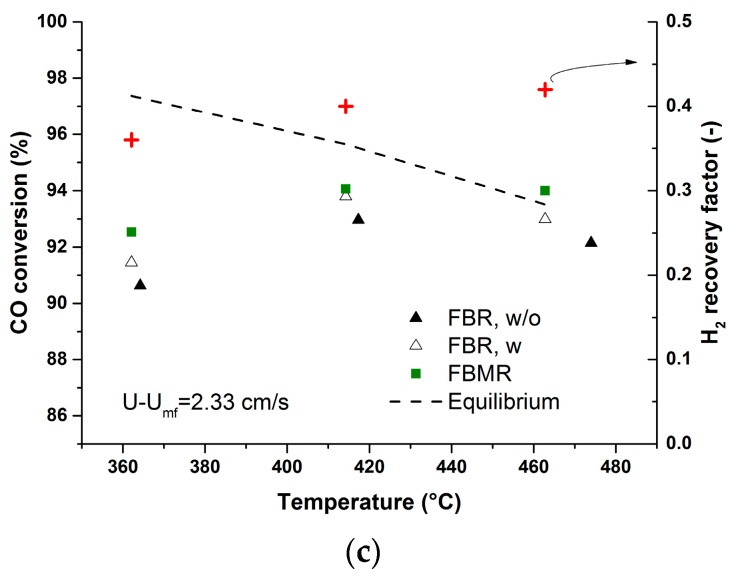
(**a**) Performance of the fluidized bed reactor without internals (FBR, w/o) in comparison with (**b**) fluidized bed reactor with internals (FBR, w) and (**c**) fluidized bed membrane reactor (FBMR) performance at various U-U_mf_. P: 1 bar, CO (10%), H_2_O (30%), N_2_ balance.

The performance of the membrane reactor was studied at different operating conditions. Firstly experiments were carried out at 1 bar inside the reactor and S/C: 3 for different excess velocities U-U_mf_: 0.77–2.32 and at different temperatures of 350, 400 and 450 °C (see Figure 6). The CO conversion of the conventional fluidized bed without internals (FBR, w/o) is considered as the reference case, which is compared to the case of a fluidized bed with internals (FBR, w) where the membrane module is just immersed but no gas is extracted via the membranes, and the case of a fluidized bed membrane reactor (FBMR) with extraction via the membranes. FBR, w/o showed higher CO conversions at elevated temperatures. The same behavior can be observed for FBR, w due to the increased catalytic activity as well. In both cases the conversion of CO is limited by thermodynamic equilibrium while only a small change in reactor performance was observed in absence (FBR, w/o) and in presence (FBR, w) of internals. This could be due to the fact that the presence of internals did not improve the mass transfer inside the bed. More studies need to be performed to figure out the optimum placement of the membrane module (bubble size *vs.* membranes pitch) to improve the mass transfer inside the bed. Possibly to have smaller average bubble size along the bed which will result in higher bubble to emulsion mass transfer rates as described by Maurer *et al.* [9].

The main advantage of utilizing hydrogen selective membrane (FBMR) to circumvent the thermodynamic equilibrium limitation is clearly shown in Figure 6a–c where at elevated temperatures, higher CO conversions than the equilibrium value can be achieved. More specifically, at elevated temperatures, the permeation through the membranes was the rate limiting factor and determined the membrane reactor performance. This also suggests to work at higher temperatures where the H_2_ recovery increases as a result of increase in membrane permeation, while at higher excess velocities the H_2_ recovery factor decreases due to lower ratio of the membrane area to inlet flow rate. This ratio is indeed one of the key parameters determining the membrane reactor performance [7].

**Figure 7:**
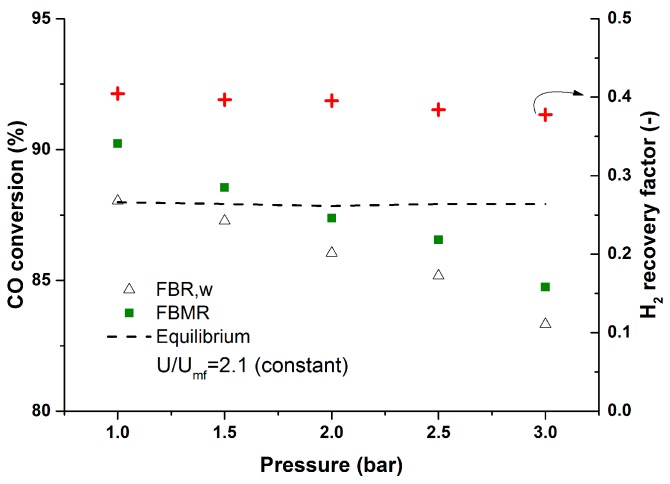
Effect of pressure on the performance of the membrane reactor at constant U/U_mf_ at 400 °C, CO (10%), H_2_O (15%), N_2_ balance, P_permeate_: 30 mbar.

During the fluidized bed membrane reactor experiments the quality of H_2_ in the permeate stream was monitored. The average CO impurity during the experiments at different temperatures was 13 ppm (min: 6 ppm, max: 25 ppm), which is very suitable for most H_2_ applications. The performance of the membrane reactor was investigated at different pressures from 1–2.5 bar. To have similar hydrodynamics inside the reactor, the inlet flow rate was modified at different temperatures to keep the U/U_mf_ = 2.1 and constant for all the cases. Figure 7 illustrates the performance of the membrane reactor at different pressures where increasing the reactor pressure will result in lower hydrogen recovery factors (average CO impurity: 21 ppm; min: 19, max: 25 ppm). This is due to the fact that at higher pressures the inlet flow rate was increased to keep the U-U_mf_ value constant for different cases. Although the hydrodynamics will be similar for the different cases, the ratio between the inlet flow rate and the membrane area is different for the different cases. Increasing the inlet flow rate at higher pressures will result in a lower ratio of the membrane area over the inlet flow rate.

**Figure 8:**
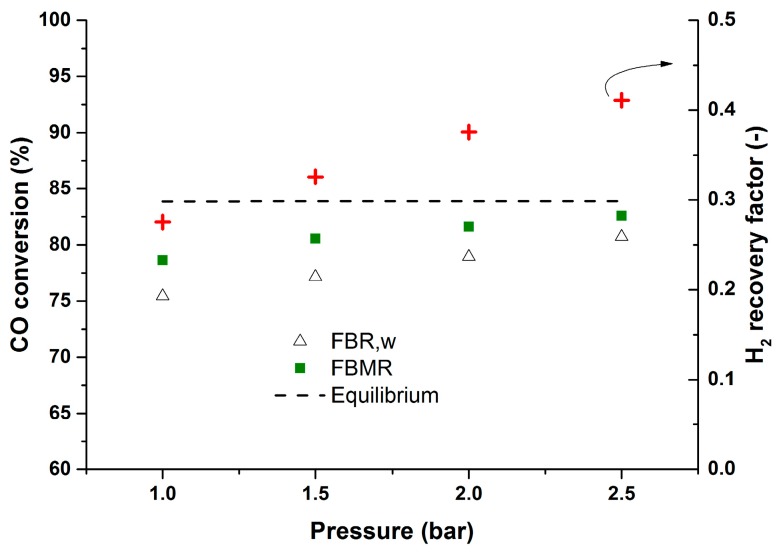
Membrane reactor performance at different pressures up to 2.5 bar and at 400 °C, CO (5%), H_2_O (15%), CH_4_ (0%), H_2_ (18%), N_2_ balance, U/U_mf_:1.71–6, P_permeate_: 30 mbar.

To show this more clearly, a new test with constant inlet flow rate was carried out for different operating pressures. Figure 8 clearly shows higher CO conversions at higher membrane reactor pressures when keeping the inlet flow rate constant. In this case at higher pressures the hydrogen recovery will be higher as well (average CO impurity: 7 ppm, min: 3 ppm, max: 9 ppm). Although increasing the pressure above 2 bar inside the reactor did not affect the performance of the membrane reactor much. This is due to the fact that at higher pressures (with constant inlet flow rate) the U/U_mf_ ratio will be lower. Therefore, at higher pressures the mixing will be worse and this will induce the mass transfer limitation inside the catalytic bed. Although CO conversions above the equilibrium can be achieved for higher inlet flow rates, due to limitations in the CO mass flow controller, experiments for higher inlet flow rates were not feasible with the specified inlet gas composition. Therefore, experiments were continued with a semi-industrial WGS inlet gas composition.

### 2.7. Reactor Performance for Industrial Inlet Composition

**Figure 9:**
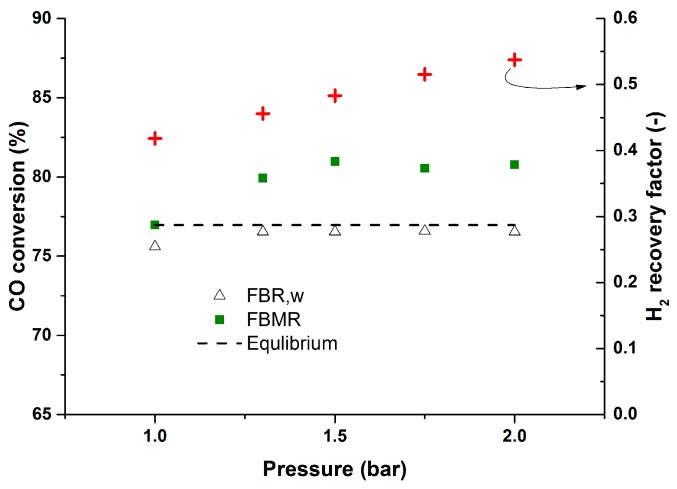
Membrane reactor performance for a semi-industrial inlet composition at 400 °C, CO (9.2%), H_2_O (19%), CH_4_ (4%), H_2_ (30%), N_2_ balance, U/U_mf_:1.67-5, P_permeate_: 30 mbar.

The membrane reactor performance was also studied by feeding a semi-industrial WGS feed composition (WGS gas as outlet of a steam methane reformer) at different pressures. The inlet flow rate was kept the same for all the cases to keep the ratio of the inlet flow rate to the membrane area constant, as in the previous study. The obtained results (Figure 9) confirmed the better performance of the membrane reactor at higher pressures, although the maximum total pressure was 2 bar due to limitations with the current setup. The CO content in the permeate is below 10 ppm (average CO impurity: 5.6 ppm, min: 4.5 max: 7 ppm), which guarantees the H_2_ quality for fuel cell applications. 

A similar experiment was carried out at 450 °C to assess whether the performance of the membrane reactor can be improved at higher temperatures. Although an increase in the temperature results in better performance of the membrane reactor in terms of CO conversion and H_2_ recovery factor, a high degree of methanation occurs at higher reactor temperatures. Apparently, the catalyst used in this study is active for methanation only at temperatures around and above 450 °C. Therefore, for the inlet composition similar to industrial WGS reactors it is recommended to operate at around 400 °C to minimize methanation.

### 2.8. Post-Mortem Analysis

#### 2.8.1. Membranes

**Figure 10:**
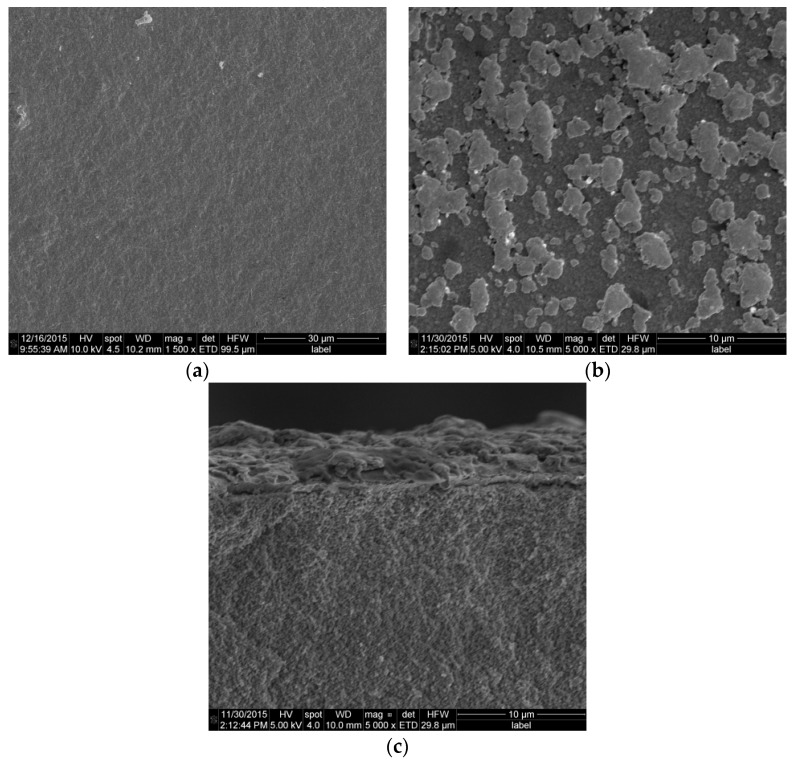
SEM images: (**a**) fresh membrane surface; (**b**) membrane surface after 900 h of continuous operation under bubbling fluidization conditions and WGS; and (**c**) membrane cross section.

**Figure 11:**
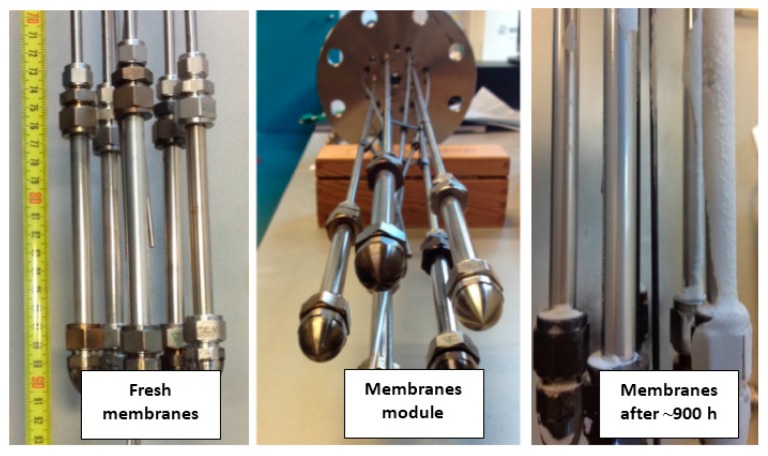
Fresh membranes surface and after 900 h of continuous operation under bubbling fluidization conditions and high temperature WGS.

After completion of membrane reactor tests the module was cooled down to room temperature and the membranes and catalyst particles were removed for further post-mortem analysis. Figure 10 and Figure 11 show the actual and SEM images of the membranes surface before and after 900 h of continuous operation. It is clear that the surfaces of the membranes are contaminated with traces of fluidizing particles. Despite the contamination with parts of fluidization particles the membrane has shown a very stable permeation throughout the entire experimental program.

#### 2.8.2. Catalyst

**Figure 12:**
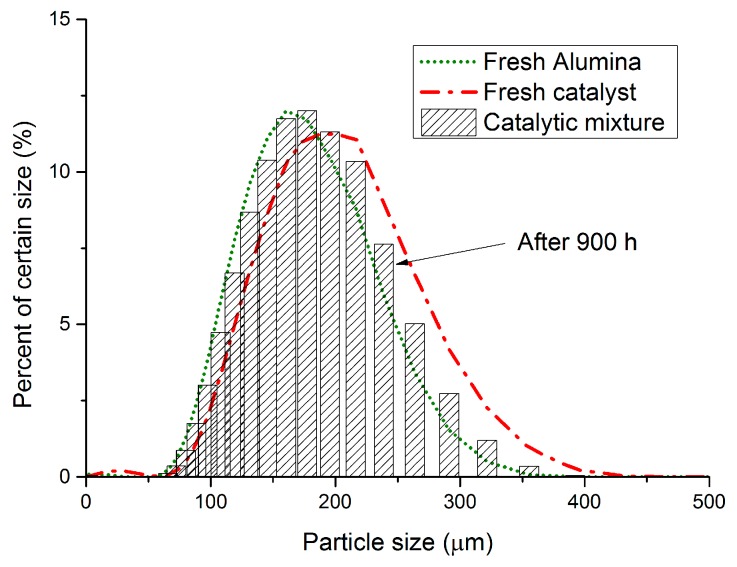
Particle size distribution of fresh alumina, catalyst and the catalytic batch after 900 h of continuous operation (FRITSCH ANALYSETTE 22).

Figure 12 compares the particle size distribution of the fresh catalyst with the particle size distribution after 900 h of continuous operation under bubbling fluidization conditions and high temperature WGS. The particle size distribution before and after the long term performance check of the fluidized bed membrane reactor module confirms the very good mechanical stability of the catalyst. Therefore, the attrition of the particles inside the column was negligible for the selected experimental conditions. Due to limitations in the setup, experiments at high inlet flow rates to attain the turbulent fluidization regime were not possible.

## 3. Materials and Methods 

**Figure 13:**
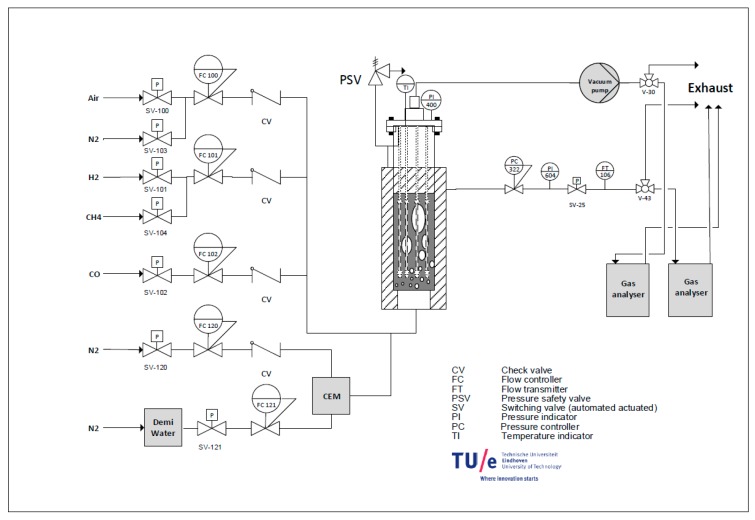
A process flow diagram of the membrane assisted fluidized bed setup.

**Figure 14:**
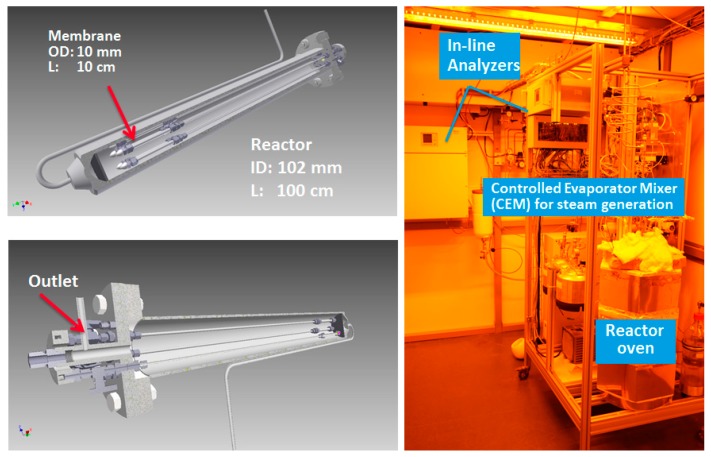
Fluidized bed membrane reactor setup (**right**); and membrane reactor module (**left**).

A membrane assisted fluidized bed reactor setup was designed and built. The setup consists of three main sections: a feed section, reactor section and analysis section (Figure 13 and Figure 14). The feed section consists of a feed gas supply from cylinders and mass flow controllers (Bronkhorst^®^ Bronkhorst Nederland B.V., The Netherlands) to set the desired flow and feed composition at the inlet. The mass flow controllers are also equipped with shut off valves (Nypro type) to cut off the flow in case of an emergency. A Bronkhorst^®^ Controlled evaporator and mixer (CEM) system was used to feed the reactor with a precise and very stable amount of steam. All the lines were electrically traced to ensure that the temperature remains above the dew point of the gas mixture.

To control the system remotely, the InTouch program (InTouch 2012 V10.6, Houston, TX, USA) was used on a PC. In the permeate side the CO content (in ppm) was continuously measured to be able to assess the stability of the membrane performance.

The reactor was constructed with a geometry of 102 mm in diameter and 100 cm in height from AISI310 (CORO METAALTECHNIEK, Veenendaal, The Netherlands). The porous plate distributor was made of Hastelloy X (Ø 102 × 5 mm) with 40 µm pore size. Three electric baby ovens with a capacity of 2.2 kW were used to keep the reactor at the desired temperature. Pressure transmitters (PTX 1400 from Druck Nederland B.V., The Netherlands) were used to measure the pressure at the top and bottom of the reactor (at the porous plate position). The feed can be set to bypass the reactor using a three-way valve (Parker type) to measure the composition with an analyzer.

The setup was designed to have the possibility to sample both the retentate and permeate streams. The analysis section consists of two inline GMS800 series extractive gas analyzers (© SICK GmbH, Reute, Germany). In the retentate side, the analyzer was calibrated for CO, CO_2_, CH_4_ and H_2_ with a precision of 1 vol%. In the permeate side the analyzer was calibrated for H_2_ between 0 to 100 volume % and for CO between zero and 100 ppm. Therefore, traces of CO impurity at the permeate side can be detected with the analyzer (with a lower detection limit of 0.2 ppm). CO_2_ is also measured in the permeate side stream in the range 0–200 ppm.

Catalyst and filler particles were supplied by Johnson Matthey^®^ (Johnson Matthey Fuel Cells Research, Reading, UK). The catalyst was 2 wt % Pt loading impregnated onto 80–200 micron alumina particles (Table 4). Due to high activity of the catalyst, it was diluted with inert alumina (the catalyst support) to have enough bed height for complete immersion of the Pd-based membrane module inside the gas-solid suspension at minimum fluidization velocity. Thus a mixture of 954 g of the catalyst and the filler (9.2%/90.8%) was integrated inside the reactor for these experiments. Separate tests with different amount of catalyst material confirmed the absence of mass transfer limitations.

Regarding the hydrodynamics of the fluidized bed, separate experimental results (not reported here for brevity) confirmed homogeneous fluidization of the catalyst and alumina filler particle mixture. The minimum fluidization velocity (U_mf_) was determined experimentally for the catalyst and inert material at the temperature range of 20–400 °C with compressed air using the standard pressure drop method (see Table 5). Later, the determined values were used to predict the inlet flow rate necessary to keep the catalyst bed at bubbling fluidization conditions.

Before integration of the catalyst batch inside the reactor a small batch of the catalyst/filler (1/3) mixture was used for a segregation test. After a 24 h test under bubbling fluidization conditions at room temperature and atmospheric pressure no segregation was observed, ensuring a homogenous catalyst distribution inside the fluidized bed during the experiments.

Five Pd-Ag alloy membranes (4–5 µm of selective layer thickness, 13%–15% Ag) were prepared onto ceramic supports provided by Rauschert Kloster Veilsdorf (alumina 100 nm pore size top layer, 10/7 mm outer diameter/inner diameter). The membranes were sealed following a sealing technique reported by Fernandez *et al.* [19]. After sealing, each membrane had an average net length of 10 cm.

Three sets of experiments were performed: (a) The catalyst batch was firstly integrated inside the reactor to be operated in absence of the membrane module (conventional fluidized bed reactor); (b) The catalyst batch was removed from the reactor and the membrane module was integrated inside the reactor. Membranes were first activated under pure H_2_ atmosphere ensuring stable performance of the membranes. Subsequently, the permeation properties of the membranes were checked and the inhibitory effect of CO and H_2_O on the H_2_ permeation flux was measured; (c) The catalyst batch was re-integrated into the reactor in the presence of the membrane module to study the reactor performance with internals (membranes were closed at the top so that the membranes acted as non-permeable internals). Finally, the membrane module was opened at the permeate side and the performance of the fluidized bed membrane reactor was investigated for different operating conditions. After the tests, the morphology of the membranes was analyzed by SEM and compared with the fresh membranes.

## 4. Conclusions

A fluidized bed Pd-based membrane reactor unit with a capacity of 1 Nm^3^/h of ultra-pure H_2_ was designed, built and operated for over 900 h of work. Initially, the permeation properties of the membranes were measured in absence of catalytic particles, confirming a very high H_2_ permeance and outstanding H_2_/N_2_ ideal perm-selectivities (up to 21,000 when integrating five membranes in the module) in comparison with the best ever reported Pd based membranes in the literature. Independent effects of external mass transfer limitations (concentration polarization), CO poisoning and H_2_O inhibition on the performance of the membranes were investigated in the single-phase module over a temperature range of 350–450 °C and for different H_2_ concentrations in the feed. The obtained results revealed that concentration polarization is the rate limiting factor for H_2_ molecules to reach the surface of the membranes and a better mixing inside the membrane reactor module is essential, which can be achieved with fluidization. In line with other findings in the open literature, the poisoning effect of CO was decreased at higher temperatures while the inhibitory effect of H_2_O was negligible over the investigated temperature range.

Monitoring the performance of the membrane reactor for a reference case over 900 h of continuous work under bubbling fluidization and high-temperature WGS conditions confirmed a very stable performance of both the membranes and the catalyst. The membrane reactor performance was studied at different operating conditions and compared with the performance of a conventional fluidized bed reactor and used to evaluate the optimal operating conditions. The fluidized bed reactor performance with and without and the membranes as internals without permeation was practically the same, indicating that the presence of the membranes tubes did not improve the bubble-to-emulsion phase mass transfer, possibly related to the suboptimal positioning of the membrane module inside the reactor. Further studies are required to optimize the positioning of the membrane module.

In general, increasing the temperature to between 350 and 450 °C results in higher CO conversions and improved H_2_ recovery factors, although at higher temperatures the performance can be deteriorated due to the increasing importance of methanation (at least for the catalyst used in this work). In addition, it is recommended to operate at higher pressures to enhance the permeation through the membranes thereby shifting the equilibrium more towards the products. Analysis of the H_2_ quality in the permeate stream has shown very low CO concentrations (in average <10 ppm), so that the produced hydrogen can be directly fed to a low temperature PEM fuel cell.

## Figures and Tables

**Table 1:** Hydrogen permeation properties of commercial and the best thin Pd based membranes (membranes reported in order of permeance, the units of the permeance have been taken from DOE 2015 [12] and used to compare the different membranes).

Membrane	Support	Thickness µm	Technique	Temp °C	Calculated Permeance × 10^−7^ mol·m^−2^·s^−1^·Pa^−1^ at 1 atm	Selectivity H_2_/N_2_	Producer	Ref.
Pd77Ag23	No	1.9–3.8	PVD 2 steps	400	190	2900	Sintef	[13]
Pd93-Ag7	α-Al_2_O_3_	0.78	ELP	400	114	640	TECNALIA	[14]
Pd77Ag23	Micro-channels	2.2	PVD 2 steps	400	88	----	Sintef	[15]
Pd92-Ag8	γ-Al_2_O_3_/YSZ	0.9	ELP	400	65	1100	TECNALIA	[16]
Pd-Au	Al_2_O_3_	2–3	ELP	500	62	1400	Dalian	[17]
Pd-Au	YSZ/PSS	1–5	ELP	400	43–52	10,000–20,000 (H2/Ar)	PALL *	[18]
Pd85-Ag15	α-Al_2_O_3_	3.2	ELP	400	31	8000–10,000	TECNALIA	[19]
Pd-Ag	α-Al_2_O_3_	2–10	ELP	350	6–31	500 to >1000	Media and process *	[20]
Pd	γ-Al_2_O_3_	2–4	ELP	400	27	500	Dalian	[21]
Pd	Metallic	3–5	ELP	450	20	450 H_2_/He	CRI/Criterion *	[22]
Pd-Ru	Al_2_O_3_/PSS	6.4	ELP	400	19	15000 @ 10 bar	NORAM * (former MRT)	[23]
Pd-Ag	α-Al_2_O_3_	3–9	ELP	350	15	>7600	Hysep *	[24]
Pd	Metallic	12	PVD-ELP	417	11	1100	Plansee/KIT/Linde	[25]
Pd85-Ag15	Metallic	4–5	ELP	400	10	>200,000	TECNALIA	[10]
Pd	Metallic	7.6		450	9	Infinite after 3500 h: >6000 H_2_/He	CRI/Criterion *	[22]
Pd	No	76	Self-supported	600	4	>>10,000	REB Research *	[6]
Pd85-Ag15	α-Al_2_O_3_	4	ELP	400	42	20,000	TECNALIA	This work

* Commercial Pd-based membranes.

**Table 2:** Overview of the operating window for the experiments.

Parameter	Unit	Value
Pressure Range	bar	1–3
Temperature Range	°C	350–450
U/U_mf_	-	1.5–5
Steam/Carbon (S/C)	-	1.5–3

**Table 3:** Parameters that were used to quantify the reactor performance.

CO Conversion	$\frac{\phi_{co,in}-\phi_{co,out}}{\phi_{co,in}}$
**H_2_ Recovery Factor**	$\frac{\phi_{H_{2},permeated}}{\phi_{H_{2},in}+\phi_{H_{2},produced}}$

φ: Molar flow.

**Table 4:** Catalyst and alumina particles physical properties.

	Material	Avg. Particle Diameter (µm) ^1^	Average Skeletal Density (g/cc) ^2^	std. dev. (g/cc)	Apparent Density ^3^ (g/cm^3^)	Geldart Classification (-)
Filler	Al_2_O_3_	160	3.300	0.009	1.691	A/B
Catalyst	2%Pt/Al_2_O_3_	180	3.321	0.016	1.443	A/B

^1^ FRITSCH ANALYSETTE22 + Quantachrome instruments, Upyc 1200e V5.04; ^3^ ThermoFisher SCIENTIFIC Pascal 140 series.

**Table 5:** Minimum fluidization velocity *vs.* temperature.

Temperature (°C)	22	102	204	296	397
U_mf_ (cm/s)	2.41	2.14	1.90	1.63	1.49
